# Stable individual differences in strategies within, but not between, visual search tasks

**DOI:** 10.1177/1747021820929190

**Published:** 2020-07-09

**Authors:** Alasdair DF Clarke, Jessica L Irons, Warren James, Andrew B Leber, Amelia R Hunt

**Affiliations:** 1 Department of Psychology, University of Essex, Colchester, UK; 2 Department of Psychology, The Ohio State University, Columbus, OH, USA; 3 School of Psychology, University of Aberdeen, Aberdeen, UK

**Keywords:** Visual search, individual differences, optimal behaviour, eye movements

## Abstract

A striking range of individual differences has recently been reported in three different visual search tasks. These differences in performance can be attributed to strategy, that is, the efficiency with which participants control their search to complete the task quickly and accurately. Here, we ask whether an individual’s strategy and performance in one search task is correlated with how they perform in the other two. We tested 64 observers and found that even though the test–retest reliability of the tasks was high, an observer’s performance and strategy in one task was not predictive of their behaviour in the other two. These results suggest search strategies are stable over time, but context-specific. To understand visual search, we therefore need to account not only for differences between individuals but also how individuals interact with the search task and context.

## Introduction

As is common in cognitive psychology, most visual search literature has focused on how the average participant performs in the task, despite it being well known that there is a great deal of variability between one subject and the next. From Treisman’s work on Feature Integration Theory (Treisman & Gelade, 1980) to the latest incarnation of the Guided Search Model (Wolfe et al., 2015), we have a good understanding of what makes particular objects easier or harder to find. However, these theories and models have neglected the question of why some observers find visual search so much harder than others. These differences can emerge from several different sources of variation: tiredness (Mackworth, 1948), information-processing ability, speed-accuracy trade-off, motivation, visual impairments (Nowakowska et al., 2016), and search strategies (Boot et al., 2006). Although their existence has previously been noted (Clarke et al., 2019; Mackworth, 1948), a rigorous examination of individual differences in visual search is a challenge that has not been taken up by many researchers, and questions about their impact and stability remain relatively underexplored.

Here, we focus on one source of individual differences in visual search: strategy. By strategy, we mean a collection of search behaviours from which all observers can freely choose. Examples include adopting a systematic left-to-right and top-to-bottom strategy (Gilchrist & Harvey, 2006), or prioritising locations that, based on knowledge or context, are more likely to contain the target (Wolfe et al., 2015). A striking example of the effect of strategy is given by Boot et al. (2006). They asked participants to monitor a cluttered display for an object changing colour or suddenly appearing. Large individual differences were found with respect to the number of saccades participants made while monitoring the stimulus, which was negatively correlated with detection performance.

**Figure 1.**
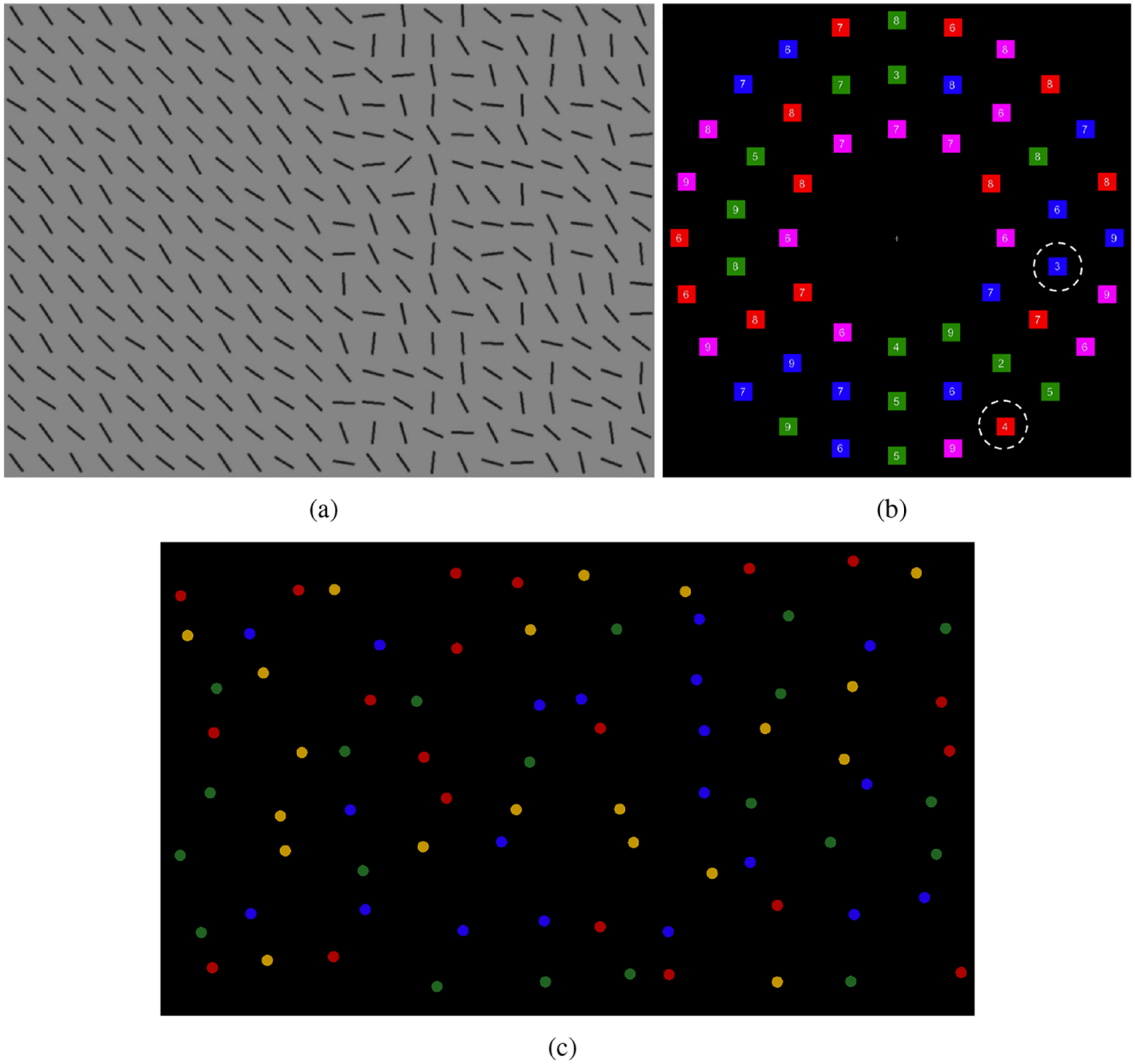
Example stimulus from the (a) *split-half line segment* (SHLS), (b) *adaptive choice visual search* (ACVS), and (c) *mouse click foraging task* (MCFT) paradigms.

Eye movement strategies have also been shown to be an important source of individual differences in visual search efficiency. Nowakowska et al. (2017) designed a simple search paradigm to discriminate between optimal (Najemnik & Geisler, 2008) and stochastic (Clarke et al., 2016) search strategies. Participants searched through arrays of line segments (Figure 1) arranged such that those on one side of the display all had a very similar orientation (homogeneous), while those on the other side had higher variance (heterogeneous). This meant that targets appearing on the homogeneous side were highly salient, while targets on the heterogeneous side were harder to find. The optimal strategy here is to search the heterogeneous half, as targets on the homogeneous side can be detected with peripheral vision. We will refer to this paradigm as the split- half line segment (SHLS) task. Some participants searched the displays near optimally, but others carried out strategies counter to this, failing to even match the performance of the stochastic searcher. The degree to which participants made saccades in line with the optimal search strategy was strongly correlated with the speed of their search. A related version of this paradigm has been used in research investigating eye movement strategies in response to (simulated) hemianopia (Nowakowska et al., 2016, 2018) with similar conclusions: the full spectrum of individual differences in strategy was observed. It is therefore not possible to conclude whether optimal or stochastic models better describe search without first explaining individual variability.

A similar range of strategies, from random to near-optimal, has been found by Irons and Leber (2016) with the adaptive choice visual search (ACVS) paradigm. This paradigm involves stimuli made up of small coloured boxes (red, blue, green, and a fourth colour that varies from red, through purple, to blue and back again) with numerals written inside them (Figure 1). The target is defined as a red or blue box containing one of four numerals (e.g., 2–5), and on each trial, one target of each colour is present. The participant’s task is to find one of either target as quickly as possible and report the numeral. On trials where the fourth variable colour is red (or close to red), participants should search through the blue boxes and report the blue target, as there will be fewer distracters. As the fourth colour changes through purple to blue, participants should update their strategy and search for the red target. The results showed that participants varied substantially along two key dimensions: how frequently they used the more effective target colour to search (varying from chance performance to near optimal) and how often they changed between colours. Further work (Irons & Leber, 2018) has shown that these differences are stable over time (between 1 and 10 days) with test–retest correlations of around r=.83 (95% confidence interval [CI] = [0.72, 0.90]) for optimal choices.

Another example of differences in search strategy comes from the foraging literature (Jóhannesson et al., 2016; Kristjánsson et al., 2014). In this context, foraging tasks involve searching for multiple targets on each trial. Participants were asked to search through a set of items from four categories, with two categories classed as targets. In the conjunction condition (searching for red-horizontal and green-vertical line segments among red-vertical and green-horizontal distracters), most observers searched in runs, finding all the targets of one target category, and then switching and finding the targets in the other category. This strategy has previously been observed in animal foraging (Dawkins, 1971) and suggests holding one complex target template in mind at a time is a better strategy than switching templates. However, a subset of participants, termed “super-foragers,” were able to change between search target categories with very little cost to performance. While test–retest reliability has not been measured explicitly for the foraging paradigm, the task was used as a measure to assess the effect of a 6-day mindfulness retreat on cognitive performance (Hartkamp & Thornton, 2017). From a re-analysis of these data, we can estimate that the test–retest reliability for the mean run length is r∼∼.7 for the feature condition and r∼∼.88 for the conjunction search.

Previous research has investigated the relationship between these behaviours and psychometrics, but to date, these differences have not shown strong correlations with other attributes. Irons and Leber (2016, 2018) found no evidence of a correlation between the proportion of optimal choices made by observers in the ACVS paradigm and measures of visual working memory, trait impulsivity, novelty seeking, need for cognition, and intolerance of uncertainty. Similarly, the differences in foraging behaviour are not accounted for by working memory or inhibitory control (Jóhannesson et al., 2017). However, there is evidence of a link between attention-deficit/hyperactivity disorder and various search behaviours (Van den Driessche et al., 2019). Furthermore, the degree to which children exhibit organised scanpaths appears to develop in tandem with executive function (Woods et al., 2013).

A common theme emerging from these studies is the observation that individual strategies vary in their degree of effectiveness or optimality. However, “visual search” encompasses a wide range of tasks, each tapping into a different aspect of behaviour (e.g., feature-based attention, information sampling). The aim of the present study is to investigate the extent to which individual differences are stable across different visual search paradigms. Does it make sense to talk about “super-searchers” who show above-average performance in a range of search tasks (analogous to the “super-recognizers” of the face-recognition literature; Russell et al., 2009)? As a secondary question, we will measure the test–retest reliability of the differences found in the SHLS paradigm, and compare it with existing estimates of reliability for ACVS and MCFT ( mouse click foraging task).

## Methods

The methods and planned analysis for this study were registered on the Open Science Framework^1^ before data collection started.

### Participants

The data from 64 participants were included in this study.^2^ Participants were compensated for their time with either course credit or £15. All participants gave informed consent. The study was approved by the University of Aberdeen Psychology Ethics Committee.

Sample size was determined in part by a power analysis and in part by counterbalancing. With n=64 participants, correlations with r>.34 between the different visual search paradigms can be detected (with α=.05 and β=.80 ) between the different visual search paradigms can be detected. The sample is therefore of sufficient size to detect relatively small correlations.

### Materials and procedures

The study consists of three paradigms from the visual search literature in which large individual differences have been found (Irons & Leber, 2016; Kristjánsson et al., 2014; Nowakowska et al., 2017). Example stimuli can be seen in Figure 1. A brief overview of each paradigm is given below, with full details in Supplementary Materials. The three tasks were completed over two sessions, approximately 1 week apart. The SHLS was run in both sessions. The order in which participants completed the tasks was counterbalanced. There are 16 different possible orders of tasks/conditions; four participants completed each order for a total of 64.

The display was presented on a 17-inch CRT monitor with a resolution of 1400×1050 (n=40) or 1600×1200 (n=24) . Stimulus generation, presentation, and data collection were controlled by MATLAB, and the psychophysics and eyelink toolboxes (Brainard, 1997; Cornelissen et al., 2002; Kleiner et al., 2007) run on a Powermac. Participants sat ∼∼47cm from the screen.

#### SHLS

Stimuli consisted of arrays of black-oriented line segments against a grey background. The target was oriented $45^{\circ }$ clockwise, while the distractor items had a random orientation with a mean of $45^{\circ }$ anti-clockwise. The variance was low $\left(18^{\circ }\right)$ on one half of the display to create a homogeneous texture, and high $\left(95^{\circ }\right)$ on the other side to create a heterogeneous texture. When the target is present on the homogeneous half, it can be easily be detected with peripheral vision, but when it is in the heterogeneous half, it is much harder to detect. This was verified in Nowakowska et al. (2017): for brief presentations viewed from the centre, detection performance was close to chance for targets presented on the heterogeneous texture, and close to ceiling for targets presented on the homogeneous texture. There were 160 trials in total, and homo- and heterogeneous sides of the display were randomly varied from trial to trial. The dominant eye position was recorded using a desktop-mounted EyeLink 1000 eye tracker (SR Research, Canada).

This paradigm was carried out twice, once in each testing session, to give us an estimate of how consistent participants are in their search strategy over time.

#### ACVS

Each search display was composed of 54 red, blue, green, and variable-coloured small squares (14 of each colour) arranged in three concentric rings around fixation (see Figure 1). Variable distractors changed colours from trial-to-trial according to a 24-trial cyclical pattern: these distractors would be red for five trials, then across a period of seven trials, they would gradually change colour from red to blue. The variable distractors would then be blue for five trials, and then gradually transition back to red.

A white digit appeared inside each square. Participants were informed that two targets—a red square and a blue square each with a digit between 2 and 5—were embedded in every search display. The two target digits were always different, to enable us to distinguish the colour of the target that had been found on each trial. The remaining red, blue, and variable squares all contained digits between 6 and 9. Green squares could contain any digit between 2 and 9. The location of the targets and distractor within the search display were randomised on each trial. Participants were only required to find one target on each trial, and they were free to search for either one.

#### MCFT

In the feature foraging condition, search displays contained small red, green, yellow, and blue circles. For half of the participants, targets were red and green circles, and for the other half of participants, targets were blue and yellow circles. Participants were asked to collect all of the targets within a trial by using the mouse to click on each target. Clicking on a target caused it to disappear from the display. If the participant clicked erroneously on a non-target, the trial immediately ended and a replacement trial began. The conjunction foraging task was the same, except search displays were composed of both circles and squares. For half of the participants, the shapes were red and green, and for the remaining participants, the shapes were blue and yellow. Targets were defined by conjunctions of colour and shape (e.g., red squares and green circles, with red circles and green squares as distractors). The assignment of targets and distractors was random for each participant. The procedure was otherwise identical to the feature foraging task.

## Results

### Replication of each task

A brief summary of participants’ behaviour is given below. More time is spent on SHLS as the test–retest validity of it has not previously been assessed. Further analysis and details can be found in the Supplementary Materials.

#### SHLS

**Figure 2.**
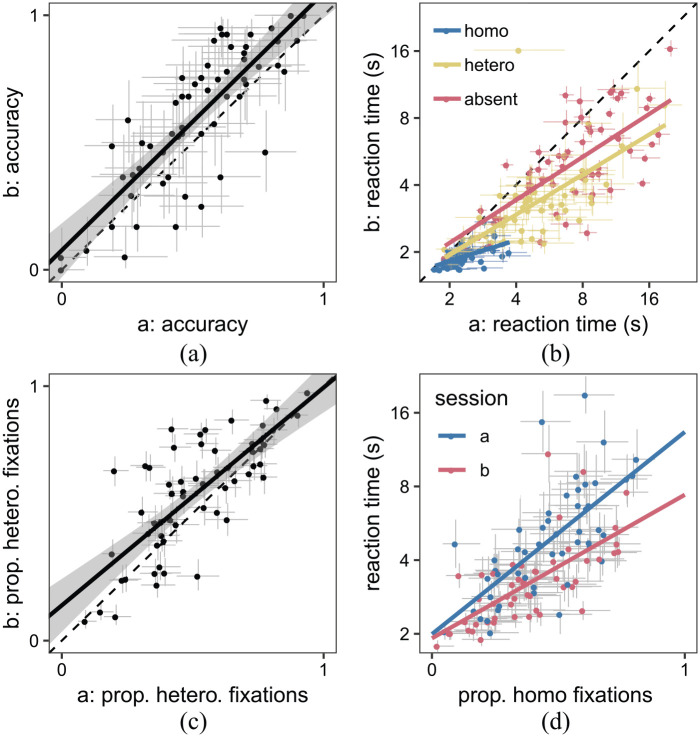
Correlation between the two sessions of the SHLS paradigm for (a) accuracy (TP-heterogeneous trials only), (b) reaction times, and (c) search strategy (TA trials only). (d) Initial search strategy correlates with reaction times in both sessions. Each point represents a participant and the error bars indicate 95% confidence intervals.

Our results are consistent with the original SHLS study (Nowakowska et al., 2017): we find a large range of individual differences in search reaction time and accuracy (see Figure 2). These differences are stable across the two sessions, with Pearson’s r∈[.71,.89] (95% CI) for accuracy in finding hard targets. We get similar scores for the correlation in reaction times between Sessions a and b for hard targets, (r∈[.54−.81]) , easy targets (r∈[.52−.80]) , and target absent trials (r∈[.66−.86]) . We can also look at the initial search strategies adopted by our participants (Figure 2c and d). Again, we see large and stable individual differences across the two sessions (test–retest r∈[.63,.86] for the proportion of the first five saccades to the heterogeneous half of the display for target absent trials). More importantly, as with Nowakowska et al. (2017), we see that the search strategies give a good correlation with reaction times in both Session a, r∈[.52,.82] , and Session b, r∈[.50,.80] .

#### ACVS

**Figure 3.**
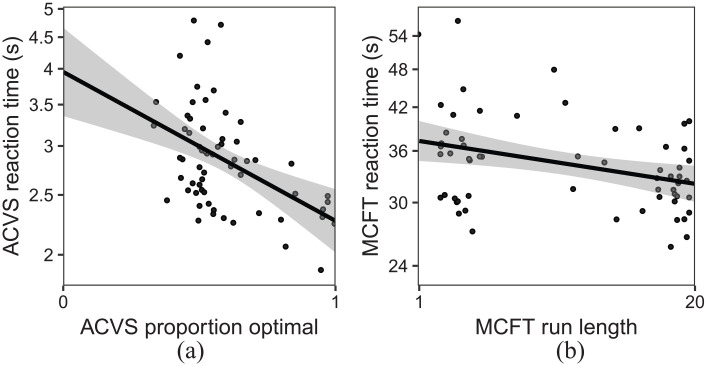
Correlation between strategy and reaction times for (a) ACVS and (b) MCFT (conjunction condition only). Each point represents a participant.

We measured an individual’s strategy as the percentage of plateau trials in which the individual chose the optimal target (i.e., the target with the fewest distractors: when the variable distractor was red, the optimal choice was blue, and vice versa). The results for the ACVS were consistent with previous findings (Irons & Leber, 2016, 2018). We can clearly see from Figure 3a that there are individual differences in the proportion of optimal targets reported (range = 33.62%–100.00%, $\bar{x}=59.15$ , s=16.54 ) and the mean $\left(\log_{2}\right)$ reaction times (range = 1.90–4.80 s). As with the SHLS task, the degree to which participants follow the optimal strategy is correlated with reaction times (r∈[−.65,−.25]) .

#### MCFT

The main measure of interest was average run length per trial in the conjunction condition, with a run defined as a succession of one or more of the same target type, which was followed and preceded by the other target or no target. The average run length was the mean number of target selections in a run. The multiple-target foraging results were in line with previous findings (Jóhannesson et al., 2016; Kristjánsson et al., 2014), with shorter run lengths for feature foraging ( $\bar{x}=3.16$ , s=3.14 ) than conjunction foraging ( $\bar{x}=11.73$ , s=7.09 ). This suggests more frequent foraging for multiple targets concurrently when those targets were defined by features than by conjunctions. Figure 3b depicts the individual differences in the conjunction condition in terms of run length and the correlation with reaction time (r∈[−.55,−.10]) .

### Correlations between tasks

We have successfully replicated the previous findings around individual differences in visual search strategy in each of the three tasks. Furthermore, the SHLS task has been shown to have good test–retest reliability, similar to that of the ACVS and MCFT tasks. Given this, we can report the extent to which an individual’s performance in one of the tasks predicts performance in the other two.

**Figure 4.**
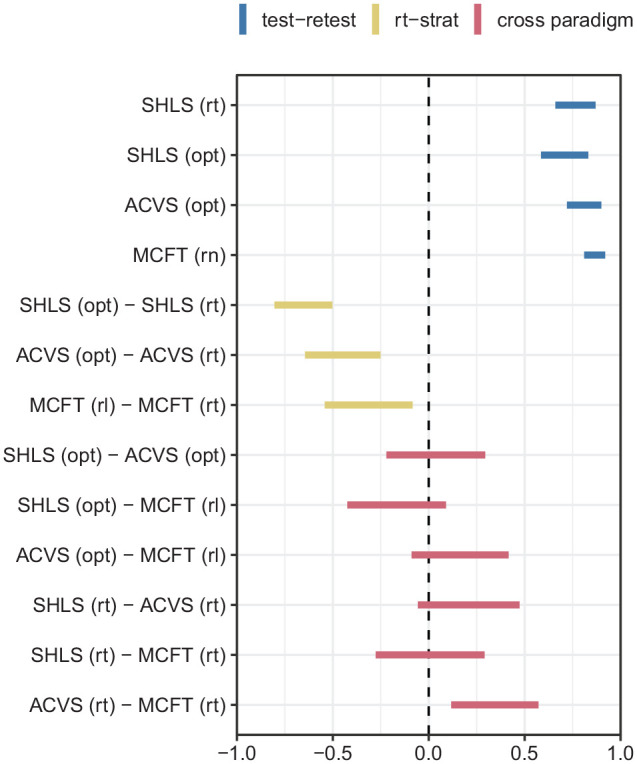
The between- and within-task correlations for the three different search tasks. The bars indicate the 95% confidence intervals for Pearson’s correlation coefficient. Blue bars represent test–retest scores for each task for reaction times (*rt*), optimality (*opt*), or run length (*rl*). Yellow bars indicate how well the strategy measures predict reaction times, while the red bars show that performance in one task is not a good indication of performance in another, either for reaction times or strategy. The *opt* measures reflect the extent to which participants adhered to an optimal strategy.

The results show that the correlations between the strategy metrics in the three tasks (Figure 4) are weak. Perhaps even more surprisingly, there is also little evidence for meaningful correlations between reaction times in the different tasks. Even if we optimistically take all data together as suggesting a robust correlation in reaction times from one task to another, the mean correlation over the three tasks is only r=.2 , implying that this correlation accounts for about 4% an individual’s performance $R^{2}=.04$ . Accuracy correlations were similarly weak; these are included in the supplementary materials.

## Discussion

We successfully replicated the wide range of individual differences in strategy and performance that had previously been observed in each of these three visual search paradigms, with a larger sample size than the original experiments. Surprisingly, however, the between-paradigm correlations give $R^{2}∼∼.04$ ; even a generous interpretation of the correlation between tasks would fail to pass the usual criteria for null hypothesis significance testing. Knowing how one person will behave in one of these paradigms tells us very little about how they will perform in the others. This lack of any consistent relationship between the search tasks occurred despite the relatively high test–retest correlations of all three of the tasks individually. Indeed, the test–retest reliability of each of the three measures of visual search strategy we used in this study compares favourably with other cognitive psychology paradigms, such as the Eriksen Flanker and Posner Cueing tasks, making them well suited for detecting relationships with other variables (Hedge et al., 2018). We also observe strong correlations between measures of strategy and reaction time *within* each task. These correlations demonstrate that our strategy metrics determine a large proportion of search performance, and that our measurements are sufficiently reliable to produce clear correlations where they exist.

There are many reasons why two measurements might be uncorrelated, such as range restriction or measurement noise, but the test–retest correlations and within-task correlations on each of the individual visual search task metrics rule out many of these alternatives, leaving a true absence of shared variance between these tasks as a likely explanation for the lack of correlation. One might have expected reaction time to be at least modestly correlated from one search task to the next, as a general factor like an individual’s speed-accuracy trade-off, or motivation might lead to better or worse overall performance, but there was no relationship. Although the tasks in this experiment all have visual search in common, they also have unique aspects that appear to have resonated with particular individuals’ strengths, and not others. Our definition of a successful strategy in the SHLS task was fixating the locations that provide new information. In the ACVS task, a successful strategy meant appropriately altering search goals to match changes in the environment. In the MCFT task, success involved minimising cognitive load by minimising target switching. Each of these tasks taps into unique aspects of visual search strategies, and performance on one has little bearing on the others. For example, recent work on the ACVS task suggests that enumerating the colour subsets plays an important role in achieving the optimal strategy (Hansen et al., 2019). Clearly, this step is not required in the SHLS search, as the stimulus consists of grey lines. Instead, participants have to judge the variability in orientation across the scene.

Individual differences pose a challenge for efforts to devise a comprehensive model of visual search. Our understanding of the mechanisms of visual search is based predominantly on experiments that systematically vary details of the search task and measure effects on average performance. This approach has led to important insights, for example, about the kinds of visual features that can guide attention (e.g., Treisman & Gelade, 1980), how attentional control settings filter distractors (e.g., Folk et al., 1992; Yantis & Egeth, 1999), and biases in attention, such as a bias towards unexplored locations (e.g., Klein, 2000). For all three of the experiments included in the current study, however, the average performance would be highly misleading, as it would describe very few of the individuals’ performance. In the original SHLS study, for example, the original aim of the experiment was to assess whether search behaviour could be better described by an optimal (Najemnik & Geisler, 2008), versus a stochastic (Clarke et al., 2016), model. Considering only the average performance, the stochastic model was a good explanation. Underlying that average performance, however, was a spectrum of search behaviour, replicated here, some of which would be clearly categorised as optimal, and some as stochastic, and some as neither. The original question needed to be refined: for whom is search optimal and for whom is it stochastic? Our approach in this experiment puts into practice several of the recommendations of Clarke et al. (2019), who suggest that a focus on accounting for variance, in addition to interpreting average patterns, will lead to important new insights. Another recommendation from that paper is to examine the generalisability of conclusions across paradigms, which we have also done here. Taking this further, it would be interesting to examine the extent to which the results of each of these search paradigms would scale to similar, but more familiar and realistic, contexts. Leber and Irons (2019) have summarised a range of methods and measures that can be used to study attention strategy (e.g., saccadic choice, speed-accuracy trade-off, metacognitive report). The current findings add even further challenges for researchers, by suggesting we need to account not only for individual differences but also for the interaction of a given individual with a particular search context.

We view these findings not as a discouraging result but as thought-provoking and exciting. Vogel and Awh (2008) argued that studying individual differences in cognitive psychology (in their case, working memory) provides valuable insight to constraining potential theories of the underlying cognitive mechanisms. Our results suggest that context and structure of the task also need to be taken into account. Understanding how an individual’s behaviour varies across different search tasks can lead to the development of a comprehensive theory of search.

## Supplemental Material

sup_mat – Supplemental material for Stable individual differences in strategies within, but not between, visual search tasks Click here for additional data file. Supplemental material, sup_mat for Stable individual differences in strategies within, but not between, visual search tasks by Alasdair DF Clarke, Jessica L Irons, Warren James, Andrew B Leber and Amelia R Hunt in Quarterly Journal of Experimental Psychology

## Notes

1. See https://osf.io/y6qbv/

2. Data from an additional 11 participants were discarded due to being recorded with an inappropriate screen resolution. Another participant was excluded due to colour blindness.
