# Supplementary material for: Stable individual differences in strategies within, but not between, visual search tasks
Source: Q J Exp Psychol (Hove). 2020 Jul 9;75(2):289–96. doi: 10.1177/1747021820929190 (PMC8721546; doi:10.1177/1747021820929190)

Supplementary Materials

Contents

Methods **1**
Split-Half Visual Search 1
Adaptive Choice 1
Foraging 2

Data Processing **2**
Participant Exclusions 2
Split-Half Visual Search task 2
Adaptive Choice 2
Foraging 2
Data Processing 4
Response Times 4
Split-Half 4
Invalid responses 4
Eye Movements 4
Accuracy 5

Methods

The experiment took part over two sessions, each lasting approximately 1 hour. All participants reported normal or corrected to normal vision and were from the University of Aberdeen. Participants were either compensated with course credit or £15 for their time. All participants gave informed consent.

All tasks were completed on a 17-inch CRT monitor with a resolution of 1400x1050 (n = 40) or 1600x1200 (n = 24). All tasks were programmed in MATLAB R2009b (version 7.9.0.529) using psychtoolbox and eyelinktoolbox.

Split-Half Visual Search

This task lasted approximately 30 minutes and was carried out in both sessions. A chin rest with forehead bar ensured a viewing distance of 47cm. Participants' fixations were tracked using a deskmounted Eylink 1000 to record eye position at 1000 Hz. Prior to every block, a 9-point calibration was carried out. Participants were instructed to state the presence, or absence, of a small target line segment that was tilted 45° to the right. The distractor line segments were arranged in such a way to create a "hard" and "easy" search side. 352 line segemets were arranged in 16 rows and 22 columns, with the easy and hard search sides being split vertically. The mean orientation of the distractors was orthogonal to the target orientation, with either a low variance (18°) which created a relatively homogenous texture ("easy" side), or a high amount of variance (95°) creating a more heterogeneous texture ("hard" side). Each line segment subtended approximately 1.6° of visual angle. For 50% of the trials, there was a target present. Participants were told to report whether the target was present or absent on each trial as quickly and as accuractely as possible by pressing either the left or right arrow key respectively.

In total, particiapnts completed 160 trials in each session, with the hetero- and homogenous side randomly varying from trial to trial.

Adaptive Choice

For this task, participants viewed the display from approximately 47cm away, but no chin rest was used to ensure a constant viewing distance. The search display consisted of two target squares (one red and one blue),

and 52 distractor squares: 12 red, 12 blue, 14 green, and 14 squares of variable colour, arranged in three rings around a fixation point. The squares subtended between ~1.1° and ~1.3° depending on the screen resolution the participant was tested at (either 1600x1200 or 1400x1050, respectively). The participants' task was to find either a blue or a red square that contained a number between 2 and 5 inclusive. On every trial, just one blue and one red square contained a number between 2 and 5. Participants were instructed to find and report one of them. To ensure we could tell which colour they found, the blue and red target squares would always have a different number inside. The distractor blue and red squares would always contain numbers between 6 and 9. The green squares contained numbers between 2 and 9. Participants were instructed to complete the task as quickly and as accurately as possible.

Before starting the task, participants were given 10 practice trials and were free to ask any questions before starting the main experiment. If a participant reported a number that was not in one of the two target boxes, a tone would sound to alert them to their mistake. After the practice trials, they would then complete 288 trials split into 4 equal length blocks (96 trials per block).

At the beginning of the experiment, there would be more red than blue squares for five trials. Then, across the next seven trials, the red squares would gradually change from red to blue, stay blue for five trials, and then change from blue to red across seven trials, stay red for five trials, and so on. The number of green squares was constant. After completing the task, participants were then asked to fill out a short three-item questionnaire asking them about their strategy and whether they noticed the gradual shift from one colour being the majority to the other.

Foraging

Viewing distance was approximately 47cm (no chin rest was used). At the beginning of each trial, participants were presented with 84 small shapes on the screen which subtended between ~0.6° and ~0.7° depending on the screen resolution the participant was tested at (either 1600$x1200*or*1400x$1050, respectively). Half of these were target items that participants were to click on, using the mouse cursor, until they had "collected" all of them (they disappeared when clicked). There were two types of search with this task. In feature search, participants were presented with circles of 4 different colours (red, blue, yellow, and green) and were instructed to click on all circles of two of the colours (e.g. click on the red and green, not the blue and yellow). In conjunction search, there were squares and circles coloured with two of the four possible colours, for example, they could be instructed to collect the blue squares and yellow circles, but to avoid the blue circles and yellow squares. In both conditions, if participants clicked on a non-target shape the trial immediately ended and re-started with 84 shapes in a new set of locations. Participants completed both of these conditions. Condition order was counterbalanced, and the colours of the targets was randomised across participants.

Before starting the experiment, participants completed five practice trials to familiarise themeselves with the task, after which they completed 20 experimental trials in each condition. This took approximately 20 minutes.

Data Processing

Participant Exclusions

Data were collected from 64 participants as originally planned. Four of these participants did not complete all parts of the experiment (either declining to participate in the second session, or could not be calibrated with the eye tracker), so four new participants were recruited to bring the total back up to 64.

Split-Half Visual Search task

Accuracy data is shown in Figure 1. From this we can see that there are a number of participants who did not perform the task to a reasonable standard: 15, 25, 47, and 48. These participants, in at least one session, either missed the majority of easy targets, or responded with false positives on the majority of target absent trials. After removing these participants, the lowest accuracy in either session was 93.6% for the easy targets, and 81.6% for target absent trials. This leaves us with 60 participants for the split-half paradigm.

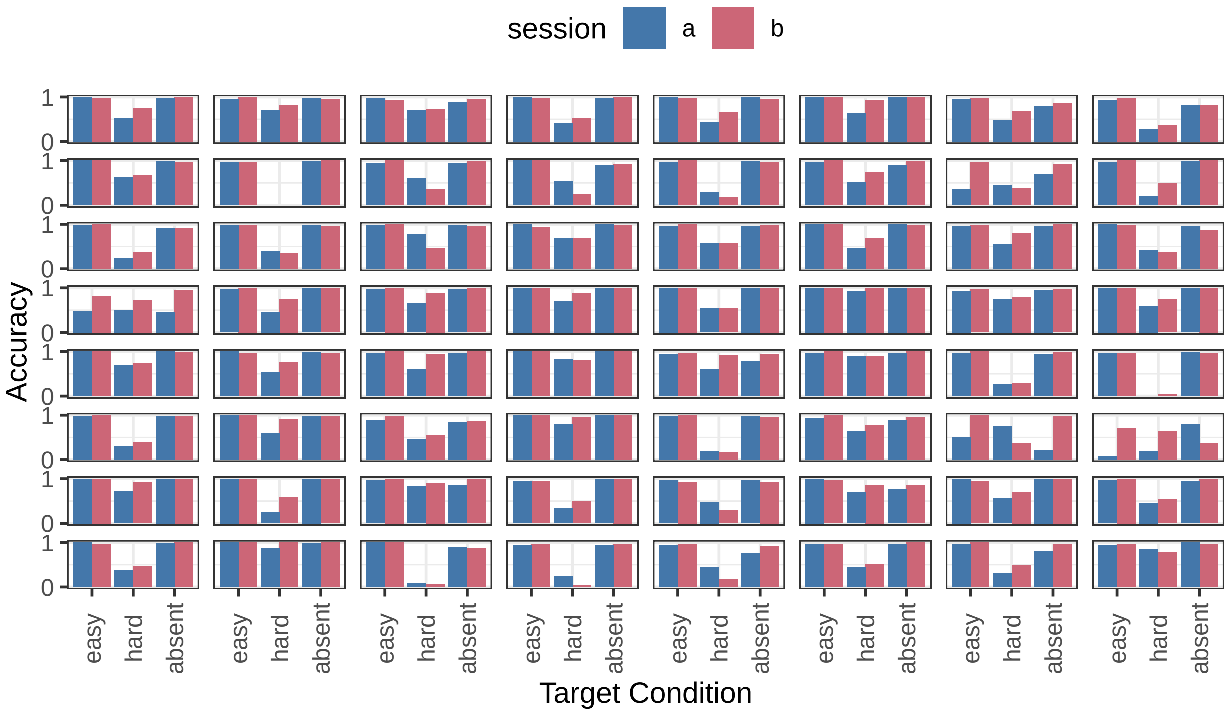

Figure 1: Accuracy data for each participant for the split-half paradigm.

Adaptive Choice

We excluded any participant with accuracy more than 3 standard deviations below the average for all participants. As can be seen in Figure 2, participants 38 & 39 were excluded for having an accuracy that was 3 standard deviations below the global average of 0.96.

Foraging

No participants were excluded for poor performance on the foraging task. However, participant 27 and participants 40 to 43 were missing their data from the feature search condition (Figure 3). We had not planned to use the results from the feature search condition in the correlation calculations, so these participants were not excluded.

Data Processing

Response Times

Response Times in the Adaptive Choice and Split-Half tasks were skewed, as can be seen in Figure 4. We therefore normalised the data by applying a log2 transformation. Response times that were 3 standard deviations from the mean were discarded.

Split-Half

Invalid responses

286 trials with invalid key responses were removed. After removing data from the four outlier participants (see above), all remaining incorrect trials (n = 2591) were removed, leaving a total of 16335 trials over 60 participants.

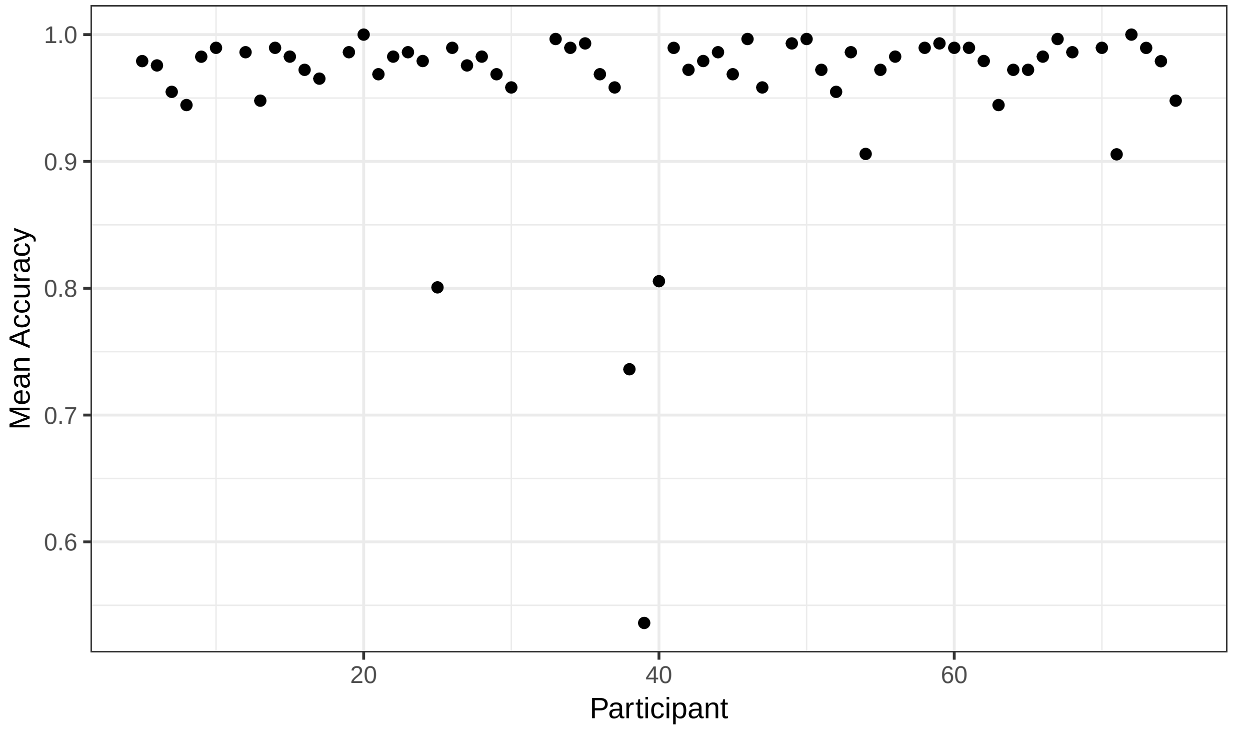

Figure 2: Mean Accuracy for each Participant in the Adaptive Choice Task

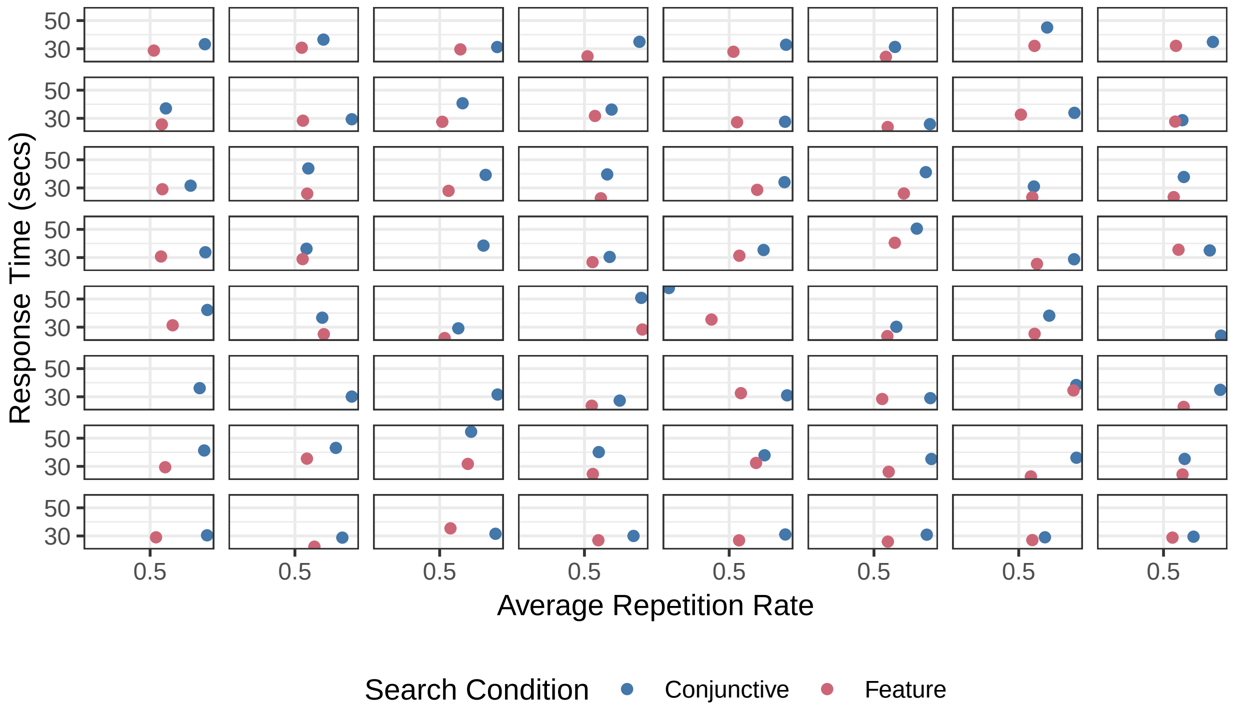

Figure 3: Average repetition rate (i.e. when the curret clicked on target is the same as the previously clicked target) and Response Times (seconds) for each participant accross conditions in the Foraging task

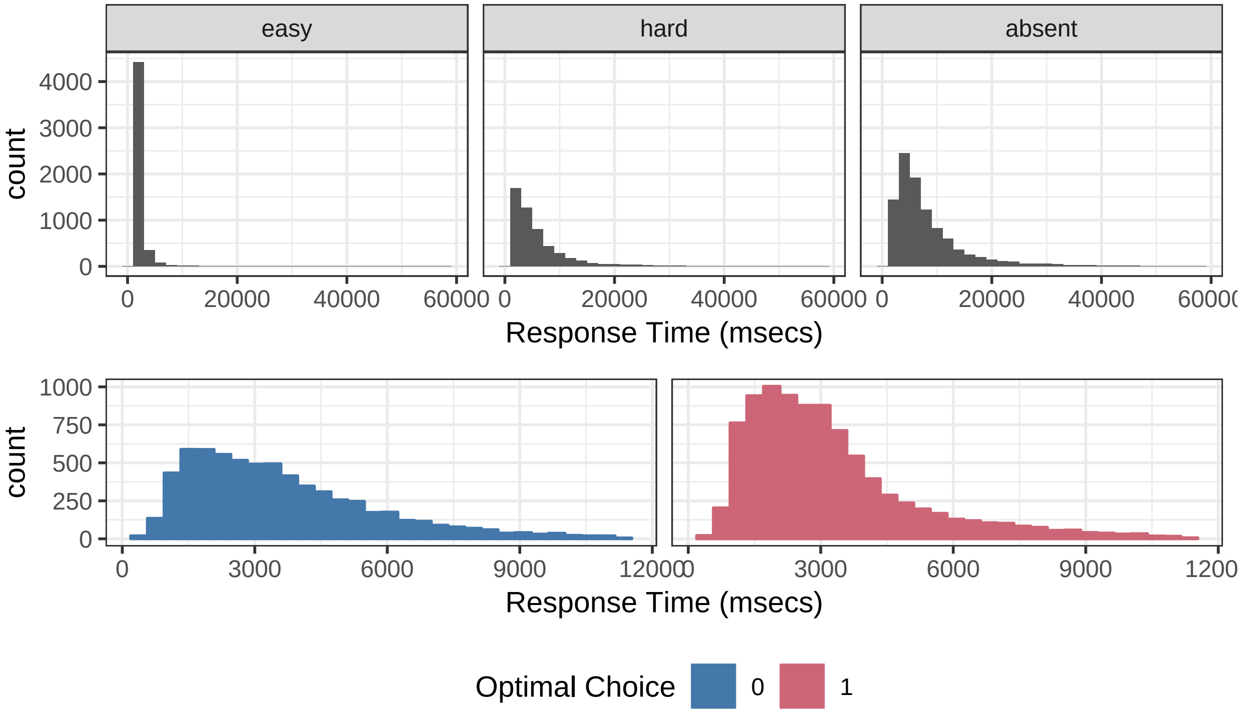

Figure 4: Response time distributions for the Split-Half (top) and Adaptive Choice (bottom) tasks. The plot for the Adaptive Choice task is split between RTs for optimal choices (i.e. picking the colour in the minority as indicated by a 1) and the suboptimal choice

Eye Movements

420564 fixations were recorded. Of these, 4283 fell outside of the stimulus area and were removed. Fixations landing within a vertical strip consisting of 10% of the stimuli's width were classed as central. All remaining fixations were then classed as landing on the homogeneous or heterogeneous half of the stimulus. Initial fixations were not included in the analysis. Numbers of fixations are given in Table 1.

Table 1: No. of fixations to each side in each condition.

targSide	fix_loc	1 < n	2 <= n <= 5
easy	central	10152	5485
easy	heterogenous	5157	5005
easy	homogenous	5736	5622
hard	central	11385	6539
hard	heterogenous	6877	6701
hard	homogenous	6783	6655
absent	central	22664	13199
absent	heterogenous	12899	12600
absent	homogenous	13651	13405

Accuracy

The results of these experiments show there is no meaningful correlation with how accurate participants were in the ACS and SHLS tasks. Even when breaking the data from the SHLS task into the 3 different trial types, there were still no meaningful correlations.

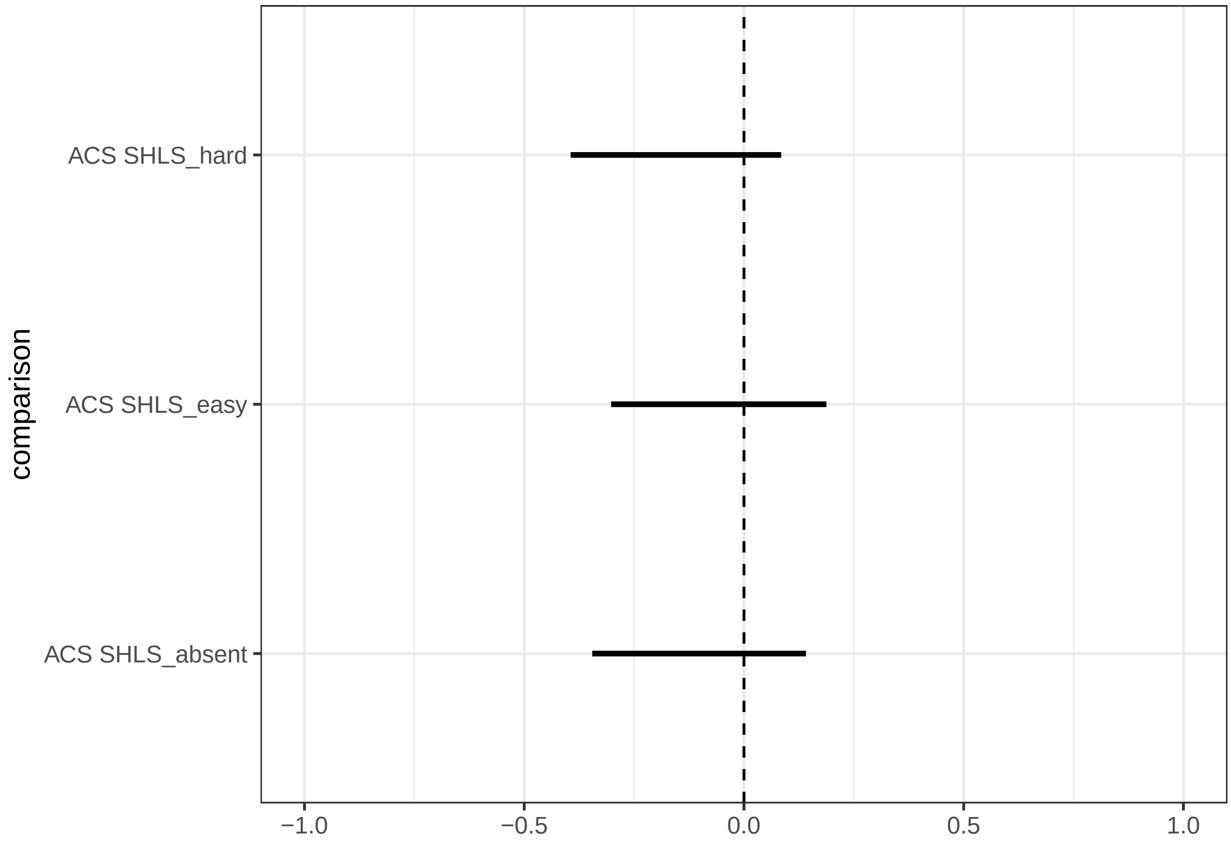

Supplement: sup_mat – Supplemental material for Stable individual differences in strategies within, but not between, visual search tasks [file sup_mat.pdf]
